# Evaluation of Self-Adhesive Composite Restorations Bond on Primary Canines: An In Vitro Study

**DOI:** 10.7759/cureus.35005

**Published:** 2023-02-15

**Authors:** Mhd Amer Orabi Kassab Bashi, Nada Bshara, Hasan Alzoubi

**Affiliations:** 1 Department of Pediatric Dentistry, Damascus University, Damascus, SYR

**Keywords:** primary canines, selective etching, microleakage, conventional composite, self-adhesive composite

## Abstract

Background and purpose

The aesthetic requirements for the restoration of the primary anterior teeth of both the child and his parents increased recently, especially with the presence of materials with good mechanical properties capable of giving a good aesthetic shape. However, the challenge remains in the possibility of applying these materials to primary teeth, where the child's cooperation and ability to keep their mouth open during the completion of the restoration is an important challenge for the dentist. Therefore, this study aimed to evaluate the bonding of self-adhesive composite by studying the microleakage in primary canines.

Materials and methods

The studied sample consisted of 60 extracted primary canines that were divided into three equal groups (n=20) according to the type of restoration: group 1 (experimental) - self-adhesive composite alone, group 2 (experimental) - self-adhesive composite with selective enamel etching, and group 3 (control) - conventional composite with the self-etching bond and selective enamel etching. A Class V cavity was prepared at the cementoenamel junction, 2 mm deep and 1 mm in diameter. In this way, the gingival wall is dentine, while the incisal wall is enamel-dentine. Restorations were placed according to the group to which the tooth belongs. After that, all teeth were subjected to 500 cycles of thermocycling. Then, a methylene blue dye microleakage test was performed, and longitudinal sections of the teeth were made and studied under x20 magnification using a stereo microscope.

Results

In the gingival wall, the scores of microleakage in the conventional composite group after selective etching were lower than those of the self-adhesive composite groups, with a statistically significant difference. While in the incisal wall, there were no statistically significant differences in the frequencies of the scores of microleakage between the three groups.

Conclusions

Within the limitations of this study, the conventional composite bonded better to enamel and dentine than self-adhesive composite resin applied alone or after selective enamel etching. The bonding of the self-adhesive composite was lower than that of the conventional composite, and it is not recommended to use it in Class V restorations of primary anterior teeth.

## Introduction

Dentists face a major challenge in treating primary teeth, especially the anterior teeth. The aesthetic requirements for the child increased during the restoration of the primary anterior teeth, but the child's cooperation and ability to keep their mouth open during the restoration completion period constitutes an important challenge for the dentist [1].

Bonding materials have undergone great developments in recent decades, as the principle of minimal preparation instigated the development of good and effective bonding materials. This process began in 1955 by Buonocore with the transition from total etch in the fourth and fifth generations to self-etch in the sixth, seventh, and eighth generations [2].

The first and second generations of bonding materials depended on the smear layer in its bonding with dentin [3], but the main problem was the inability of the bonding material to bond with the dentin, as it bonds with calcium in the smear layer and this caused weak bonding forces [4].

In the third generation, the smear layer was completely modified or removed using acid etching to allow the bonding materials to penetrate the dentinal canals and to achieve micro-mechanical bonding, and the bonding forces with the dentin reached 8-10 Mpa [5]. At the beginning of the nineties, bonding materials developed with the advent of the fourth and fifth generations. The two generations relied on completely removing the smear layer, which is called the full etching technique for both enamel and dentin [6].

Subsequently, the self-etching technique appeared, which is characterized by ease of application and shorter working time due to the shortness of clinical procedures when applied, and it also reduces postoperative sensitivity. Self-etching systems have been classified into two types: two-step bonding systems (sixth generation) and one-step bonding systems (seventh and eighth generations) [7,8].

Bulk fill restorations have become popular these days due to the shorter of time and fewer steps required for their application, which paved the way for the self-adhesive composite [9]. Self-adhesive composites consist of the traditional system of methacrylate plus the same acidic monomers in self-etch bonding materials (2-hydroxyethyl methacrylate monomer [HEMA] in addition to other monomers such as glycerol phosphate dimethacrylate [GPDM], 4-methacryloxyethyl trimellitic acid [4-MET] or bis[2-(methacryloyloxy) ethyl] phosphate [BMEP]) [10].

The first self-adhesive composite was introduced in 2009. These materials are mainly indicated for class I and class V cavities and cervical non-carious lesions, in addition to being used as pits and fissure sealants [11]. The self-adhesive composite is characterized by its low viscosity, as its fluid structure helps it to enter into the prepared cavity and thus achieve a good fit [12]; eliminating bonding systems made restoration easier to apply, saved curing time, and reduced bond application mistakes [13].

Therefore, this study was conducted to evaluate of microleakage of self-adhesive composite applied alone or after selective enamel etching compared with conventional composite applied after selective etching of enamel and self-etch bond.

## Materials and methods

Ethical consideration and sample collection

A comparative in vitro study was proposed to compare the microleakage of the self-adhesive composite compared with the conventional composite in the restoration of class V cavities in primary canines. The study protocol was approved by the Scientific Research and Postgraduate Board of Damascus University Ethics Committee of Damascus University, Damascus, Syria (IRB number: UDDS-2717-02082021/SRC-594).

The sample size was determined using a sample size calculation program (PS Power and Sample Size Calculation program, version 3.0.43). The sample size calculation produced a required sample size of 60 primary canines to detect a significant difference (90% power and two-sided 5% significance level).

Randomization

Each of the studied teeth was given a number from one to 60; then the teeth were randomized (using http://www.randomization.com) into three groups: group 1 (experimental, n=20) - self-adhesive composite, group 2 (experimental, n=20) - self-adhesive composite with selective enamel etching, group 3 (control, n=20) - conventional composite. A single-blind method was also chosen for this study because the assessors were unaware of the restorative materials used. 

Inclusion criteria

Inclusion criteria were the primary canines intact and free of caries, free of developmental and morphological defects, and free from any fractures or cracks. At least one-third of the root must be intact and not resorbed, while the teeth in which the pulp was exposed after preparing the cavity were excluded.

Work procedure

The work was conducted at Damascus University, Faculty of Dentistry, Department of Pediatric Dentistry, where the instructions of the International Organization for Standardization (ISO 11405, 2015) have been adopted with some minor modifications to suit primary teeth according to the following.

The teeth were washed with running water immediately after the extraction, and the attached soft tissues were removed. The teeth were then preserved in plastic containers containing 0.5% chloramine T solution to sterilize them for one week as a maximum, then the teeth were transferred to other plastic containers containing distilled water and kept at 4° C for a period not exceeding six months. The distilled water was replaced weekly until the start of the study.

A class V buccal cavity was prepared at the cementoenamel junction by using a high-speed round diamond bur with a water-cooled handpiece so that the gingival wall of the cavity at the cement-enamel junction is dentin and the incisal wall is enamel-dentine. The depth of the cavities was 2 mm with a diameter of 1 mm. Then the teeth were put back into distilled water and restored according to each group.

For the self-adhesive composite group, Fusio™ Liquid Dentin (Pentron, Wallingford, US) was applied in a layering technique so that the thickness of one layer did not exceed 2 mm; after waiting for 10 seconds, each layer was light cured for 20 seconds.

For the self-adhesive composite and selective enamel etching group, the enamel was etched only with phosphoric acid 37% (Ultra-Etch®, Ultradent Products Inc., South Jordan, US) for 15 seconds. Fusio™ Liquid Dentin (Pentron, Wallingford, US) was also applied in a layering technique so that the thickness of one layer does not exceed 2 mm; after waiting for 10 seconds, each layer was light cured for 20 seconds.

For the conventional composite group, the enamel was etched with phosphoric acid 37% (Ultra-Etch®, Ultradent Products Inc., South Jordan, US) for 15 seconds, then the self-etch bond (Peak SE, Ultradent Products, Inc., South Jordan, US) was applied. After that, the conventional flowable composite (TG, DMG, Hamburg, Germany) was applied in a layering technique so that the thickness of one layer did not exceed 2 mm; then, each layer was light cured for 20 seconds.

After that, the teeth were kept in two plastic containers in distilled water at a temperature of 4° C for 24 hours. Then all teeth were allocated for 500 cycles of thermocycling at 5±2°C to 55±2°C, with a dwell time of five seconds.

Microleakage studying

The apical foramen of all teeth was sealed with adhesive wax; then, the teeth were coated with two layers of nail polish except the restoration with an amount of 1 mm around its circumference, and the teeth were left to dry. Each group was immersed in methylene blue 1% in a plastic package, and the packages were closed for 24 hours. After that, they were washed well under running water to remove the remnants of methylene blue.

**Figure 1:**
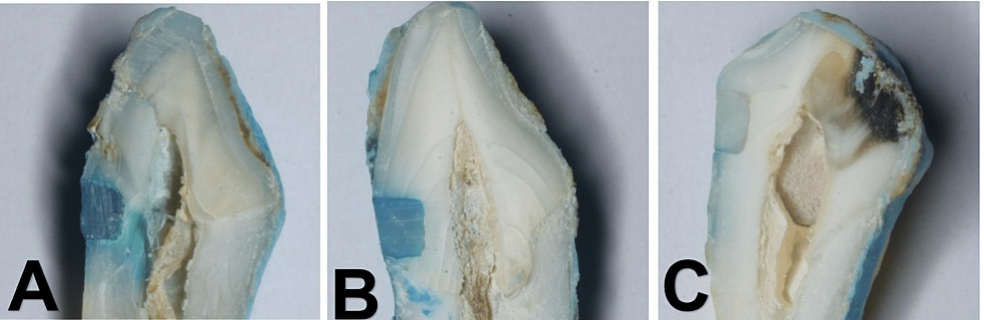
Microleakage (dye penetration) in the various restorations used in this study (A) self-adhesive composite, (B) self-adhesive composite with selective enamel etching, (C) conventional composite

Then the coronal section of each tooth was cut longitudinally into two parts in the buccal and palatal direction with a disc, which gave an evaluation surface in the mesial part and in the distal part. They were photographed using a Nikon D7200 DSLR camera with x20 magnification using a stereo microscope. The images were entered into the computer to assess the microleakage of the three groups by three external assessors as follows [14] (Figure 1). In the incisal cavity wall, score 0 was given when no dye penetration was seen, score 1 was given if the dye penetration was restricted to the enamel, score 2 was given if the dye penetrated the dentin-enamel junction and did not reach the pulp wall, and score 3 was given if the dye penetrated into the pulp wall. In the gingival cavity wall, score 0 was given when there was no dye penetration, score 1 was given if the dye penetration was less than half the thickness of the dentin, score 2 was given if the dye penetration was more than half the thickness of the dentin and did not reach the pulp wall, and score 3 was given if the dye penetrated into the pulp wall.

Statistical analysis

Statistical analysis was performed by using the SPSS 21.0 software (IBM Inc., Armonk, US). The data were analyzed with the Kruskal-Wallis test. The testing was performed at α=0.05 (p<0.05).

## Results

The studied sample consisted of 60 extracted primary canines, which were divided into three groups according to the restoration (self-adhesive composite, self-adhesive composite with selective enamel etching, and conventional composite). To study the differences in the microleakage within the incisal and gingival cavity walls between the three groups, the Kruskal-Wallis test was applied (Table 1).

**Table 1:** Basic sample characters and Kruskal-Wallis test results * indicates a statistically significant difference Group 1: self-adhesive composite alone; group 2: self-adhesive composite with selective enamel etching; group 3: conventional composite

Studied area	Groups	Score 0	Score 1	Score 2	Score 3	Rank Mean	Chi value	p-value
Incisal walls	Group 1	15%	0%	30%	55%	35.78	4.153	0.125
	Group 2	10%	10%	50%	30%	30.60		
	Group 3	45%	15%	0%	40%	25.13		
Gingival walls	Group 1	15%	25%	15%	45%	38.13	11.558	0.003^*^
	Group 2	10%	10%	15%	65%	32.50		
	Group 3	65%	0%	10%	25%	20.88		

Table 1 shows that there are no statistically significant differences in the microleakage scores between the three groups in the incisal cavity walls (p=0.125), while a statistically significant difference was found in the gingival cavity walls (p=0.003), and for pairwise comparisons, the Mann-Whitney U test was applied as shown in Table 2.

**Table 2:** Mann-Whitney U test for pairwise comparison microleakage scores in the gingival cavity walls * indicates a statistically significant difference Group 1: self-adhesive composite alone; group 2: self-adhesive composite with selective enamel etching; group 3: conventional composite

Groups		U-value	p-value
Group 1	Group 2	156.0	0.190
	Group 3	116.0	0.016^*^
Group 2	Group 3	91.5	0.001^*^

Table 2 shows that there are no statistically significant differences in the microleakage scores between the self-adhesive composite group and the self-adhesive composite with the selective enamel etching group (p=0.190). A statistically significant difference was found between the self-adhesive composite group and the conventional composite group (p=0.016) and between the self-adhesive composite with selective enamel etching group and the conventional composite group (p=0.001).

## Discussion

Microleakage is a major problem and is the main cause of composite restoration failure. Despite the large number of studies measuring the microleakage of conventional composite resin restorations, the number of studies measuring the microleakage of self-adhesive composite resins is small [9,15]. Therefore, this study aimed to evaluate the microleakage of the self-adhesive composite restorations in primary canines.

The study was conducted by preparing class V buccal cavities that are similar in shape, dimensions, and location as much as possible to unify standards. The cavities were prepared at the cementoenamel junction, where the incisal wall enamel-dentine and the gingival wall are dentin only, to test the differences in microleakage, if any, at the level of enamel and dentin [16].

The teeth were exposed to thermocycling after applying the restorations, where the teeth were subjected to 500 cycles of thermocycling to simulate the conditions within the oral cavity [17,18]. The self-adhesive flowable composite was chosen, due to the presence of studies on permanent teeth that proved that it has less microleakage, so it was necessary to study it on primary teeth [19].

Selective enamel etching has been suggested before applying the self-etching bond because there are many studies indicating that it improves bonding with the enamel [20,21]. Microleakage was investigated by penetration of methylene blue, which is an indication of incomplete sealing. Improper sealing in the oral environment can lead to microleakage, leading to the development of caries and, thus, fail the restoration procedure, and the study of microleakage gives an idea of the strength of the bond between the restoration material and the tooth structure [22].

In the gingival wall, this study showed the superiority of the conventional composite group over the self-adhesive composite applied alone and the self-adhesive composite with selective enamel etching, while there were no statistically significant differences between the self-adhesive composite applied alone and self-adhesive composite with selective enamel etching. In the incisal wall, there were no statistically significant differences between the self-adhesive composite applied alone, the self-adhesive composite with selective enamel etching, and selective enamel etching. The success is mainly attributed to the acid etching process because the hybrid layer would be stronger, thus minimizing dye penetration.

This study corresponds with the study done by Poitevin et al. and Peterson et al., which showed that the bonding forces in the self-adhesive composite group are lower than in the conventional composite group [19,23]. This study also agreed with the study done by Çelik et al., as it indicated the failure of 27 out of 40 restorations in the self-adhesive composite resin group (67%), while the success rate of restorations in the conventional composite restorations was 100% [24].

The results of this study also correspond with a systematic review conducted by Troconis and Pérez [25], while the results of this study differed from the study of Sachdeva et al., which showed no significant differences in terms of the microleakage because they did not perform thermocycling before applying the dye penetration [26].

The microleakage was assessed by applying 500 thermocycling. This is a major limitation of this study because the clinical conditions are more complicated. Additionally, there were no comparisons between the microleakages at different thermocycling.

## Conclusions

Within the limitations of this study, the conventional composite bonded better to enamel, and dentine than self-adhesive composite resin applied alone or after selective enamel etching, and selective etching before the application of self-adhesive composite did not improve the bond to enamel. The self-adhesive composite is not recommended to use in class V restorations of primary anterior teeth.
